# *Arx *and *Nkx2.2 *compound deficiency redirects pancreatic alpha- and beta-cell differentiation to a *somatostatin*/*ghrelin *co-expressing cell lineage

**DOI:** 10.1186/1471-213X-11-52

**Published:** 2011-08-31

**Authors:** Simon Kordowich, Patrick Collombat, Ahmed Mansouri, Palle Serup

**Affiliations:** 1 Department of Molecular Cell Biology, Max-Planck Institute for Biophysical Chemistry, Am Fassberg, D-37077 Göttingen, Germany; 2 Diabetes Genetics team, Inserm U636, FR-06108 Nice, France; 3 Université de Nice Sophia-Antipolis, FR-06108 Nice, France; 4 Department of Clinical Neurophysiology, University of Göttingen, Robert-Koch Strasse 40, D-37075 Göttingen, Germany; 5 Department of Developmental Biology, Hagedorn Research Institute, Niels Steensensvej 6, DK-2820 Gentofte, Denmark

**Keywords:** Arx, Nkx2.2, somatostatin, ghrelin, Pax6, Pax4

## Abstract

**Background:**

Nkx2.2 and Arx represent key transcription factors implicated in the specification of islet cell subtypes during pancreas development. Mice deficient for *Arx *do not develop any alpha-cells whereas beta- and delta-cells are found in considerably higher numbers. In *Nkx2.2 *mutant animals, alpha- and beta-cell development is severely impaired whereas a ghrelin-expressing cell population is found augmented.

Notably, *Arx *transcription is clearly enhanced in *Nkx2.2*-deficient pancreata. Hence in order to precise the functional link between both factors we performed a comparative analysis of *Nkx2.2/Arx *single- and double-mutants but also of *Pax6*-deficient animals.

**Results:**

We show that most of the ghrelin^+ ^cells emerging in pancreata of *Nkx2.2*- and *Pax6*-deficient mice, express the alpha-cell specifier Arx, but also additional beta-cell related genes. In *Nkx2.2*-deficient mice, Arx directly co-localizes with iAPP, PC1/3 and Pdx1 suggesting an Nkx2.2-dependent control of *Arx *in committed beta-cells. The combined loss of *Nkx2.2 *and *Arx *likewise results in the formation of a hyperplastic ghrelin^+ ^cell population at the expense of mature alpha- and beta-cells. Surprisingly, such *Nkx2.2^-/-^Arx^- ^*ghrelin^+ ^cells also express the somatostatin hormone.

**Conclusions:**

Our data indicate that Nkx2.2 acts by reinforcing the transcriptional networks initiated by Pax4 and Arx in early committed beta- and alpha-cell, respectively. Our analysis also suggests that one of the coupled functions of Nkx2.2 and Pax4 is to counteract *Arx *gene activity in early committed beta-cells.

## Background

The pancreas is comprised of acinar and duct cells (exocrine compartment), producing and transporting digestive enzymes and bicarbonate, as well as endocrine cells, which secrete hormones to the blood stream. The latter are aggregated into scattered clusters of cells, termed islets of Langerhans. These are typically composed of five cell subtypes, including alpha-, beta-, delta-, epsilon- and PP-cells that produce the hormones glucagon, insulin, somatostatin, ghrelin, and pancreatic polypeptide (PP), respectively. Insulin and glucagon are secreted co-ordinately to control glucose homeostasis, whereas somatostatin and PP regulate the secretion of other hormones and of exocrine enzymes [1-5]. The hormone ghrelin has been shown to possess orexigenic properties and to play a role in glucose-stimulated insulin secretion [6,7].

During pancreas development, the first wave of endocrine cell differentiation begins at embryonic day (E) 9 and results in the emergence of glucagon-producing cells, followed by the appearance of few insulin-producing cells, often co-expressing glucagon [8-12]. Ghrelin-expressing cells can be detected from E10.5 on [2,13]. Subsequently, the major wave of endocrine cell formation ("secondary transition"), initiates around E13 and results in the emergence of numerous insulin-producing beta-cells and glucagon-producing alpha-cells. Around E15, the first somatostatin-producing delta-cells emerge [10] while, shortly before birth, PP-expressing PP-cells differentiate. Concurrently, from E14 on, the endocrine cell mass continuously expands and self-organizes into well-shaped islets, a process lasting until approximately 4 weeks *postpartum *[14].

A number of transcriptional regulators have been shown to have crucial roles in controlling the specification of pancreatic cells. Among these, Pdx1 was found to exert a key function for the pancreatic cell lineage allocation. Later during pancreatic development, Pdx1 activity is restricted to beta-cells and a subpopulation of delta-cells [9,15-17]. The expression of the basic helix-loop-helix transcription factor Ngn3 induces endocrine differentiation, as evidenced by gain- and loss-of-function studies as well as genetic lineage tracing [18-21]. The specification towards the distinct endocrine cell types was found controlled by the concerted activities of specific transcription factors, including the homedomain-containing factors Nkx2.2 [22], Nkx6.1 [23], Pax6 [24], Brn4 [25], Pax4 [26] and Arx [27].

Nkx2.2 is broadly expressed in the early undifferentiated pancreatic epithelium and in endocrine progenitors. Along with the secondary transition, Nkx2.2 expression becomes restricted to mature beta-cells and a subset of alpha- and PP-cells, whereas it is not detected in delta-cells (reviewed in [28]). Accordingly, mice depleted in *Nkx2.2 *display a complete beta-cell loss and reduced alpha- and PP-cell content, while the numbers of delta-cells remain unchanged. Notably, the ghrelin^+ ^cell population appears considerably augmented [13,22]. In this context, it has been shown that Nkx2.2 is decisive for the maintenance of normal beta-cell function and that the transcriptional repressor activity of Nkx2.2 is required for the proper specification of alpha-cells [29,30]. Nkx6.1 has been found essential for the specification of multipotent progenitors towards the duct-/endocrine-fates in early pancreas development [31]. Post second transition, Nkx6.1 becomes exclusively restricted to developing beta-cells; at that stage it is essential for their expansion [23]. The Pax gene family member, Pax6, is produced in all endocrine cells throughout pancreas development with one exception that is a subpopulation of epsilon-cells [13,24]. Thus, in the absence of functional Pax6, alpha-, beta-, delta- and PP-cells are negatively affected with a near complete loss of glucagon-expression from alpha-cells [24,25,32]. Similar to Nkx2.2 mutants, the endocrine portion in Pax6^-/- ^pancreata mainly consists of cells expressing the ghrelin-hormone [2]. Pax4, another Pax family member, has been found essential for the formation of beta- and delta-cells [26,33]. Lineage tracing studies revealed that Pax4^+ ^precursor cells give rise to all four major endocrine cell types (beta-, alpha-, delta- and PP-cells) [34]. Nevertheless, in the absence of *Pax4*, beta- and delta-cell development is impaired and precursor cells acquire an alpha-cell-like fate, suggesting a predominant role of Pax4 in the differentiation of beta-cells [26,27]. This assumption is supported by recent work demonstrating that the *in vivo *misexpression of *Pax4 *in pancreatic/endocrine progenitors results mainly in the formation of beta-cells. Moreover, the forced and ectopic expression of *Pax4 *in mature alpha-cells was found sufficient to convert these into functional beta-like cells [35].

Mice lacking the aristaless homeobox gene *Arx *display a phenotype opposite to the one observed in *Pax4^-/- ^*animals [27]. Depleted in alpha-cells, such mice exhibit proportionally enlarged beta- and delta-cell populations. In fact, a direct and mutually antagonistic transcriptional repression between Arx and Pax4 was previously demonstrated and associated with the specification of the alpha- vs. beta-/delta-cell fates, respectively [36]. This model was further supported by the forced expression of *Arx *in pancreatic/endocrine progenitors, which led to the favouring of the alpha- but also PP-cell fate, at the expense of beta- and delta-cell lineages [37].

Interestingly, recent analyses of *Nkx2.2*-deficient pancreata outlined a 5-fold increase in the transcript content of the alpha-cell "specifier" Arx despite an almost complete loss in glucagon-expressing cells [38]. To address this apparent paradox, we undertook a thorough comparative analysis of the endocrine populations arising in pancreata of wild type, *Nkx2.2*- and *Pax6*-deficient animals. Moreover, we further analysed the roles of Nkx2.2 and Arx, by characterizing pancreatic endocrine cell fates in mice compound-deficient for *Nkx2.2 *and *Arx*.

## Results

### Expression of *Arx *and *Brn4 *in E16.5 pancreata of wild type, *Nkx2.2*^-/- ^and *Pax6*^-/- ^mice

We previously investigated the antagonistic roles of *Arx *and *Pax4 *for proper endocrine cell allocation and thereby demonstrated the essential function of *Arx *in promoting a subset of endocrine precursor cells specifically towards the alpha-cell fate [27,36,37]. The apparent discrepancy between the severely reduced alpha-cell numbers and the 5-fold augmentation in *Arx *transcripts upon loss of *Nkx2.2*, prompted us to examine the subcellular localization of Arx in pancreata of such mice and thus gain further insights into the role of Arx and Nkx2.2 during endocrine cell specification.

**Figure 1:**
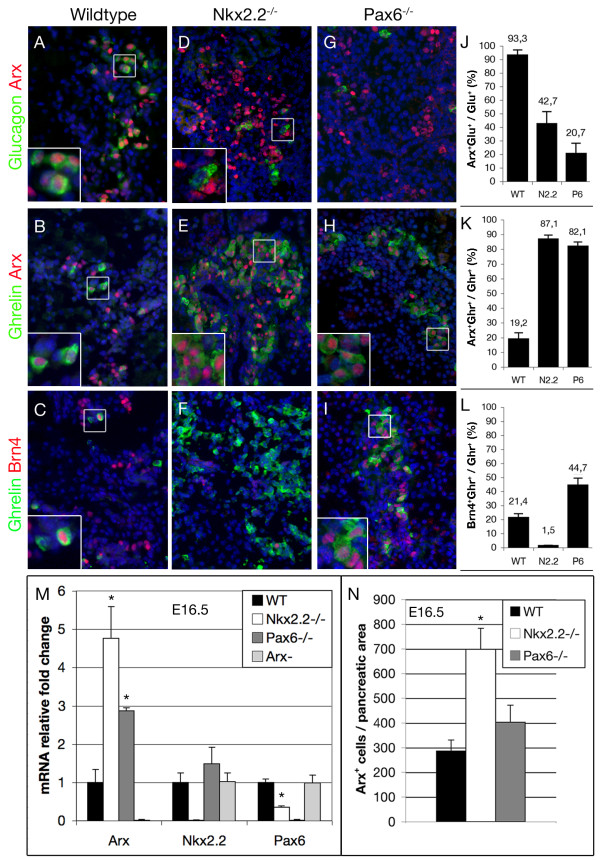
**Expression of *Arx *and *Brn4 *in E16.5 pancreata of wild type, *Nkx2.2*^-/- ^and *Pax6*^-/- ^mice**. (A, B) Arx is expressed in wild type glucagon^+ ^cells. (A, inset) Moreover, Arx^+^/glucagon^- ^cells are detectable at this stage. (B, C, inset) Arx or Brn4 can be detected in a subset of ghrelin^+ ^cells. (D, inset) In *Nkx2.2*-mutant pancreata some of the remaining glucagon^+ ^cells do not express Arx. (E, inset, F) Most *Nkx2.2*-deficient ghrelin^+ ^cells express Arx but only rarely Brn4. (G, H) Arx is expressed in pancreata of *Pax6*^-/- ^mice and co-localizes with most ghrelin^+ ^cells. Scattered Arx^+^/ghrelin^- ^and ghrelin^+^/Arx^- ^cells can be detected. (I, inset) Brn4 is expressed in a subset of ghrelin^+ ^cells and labels a ghrelin^- ^cell population in *Pax6*-deficient mice. Counterstaining with DAPI. (J-L) The percentages of Arx^+^/glucagon^+^, Arx^+^/ghrelin^+ ^and Brn4^+^/ghrelin^+ ^cells in pancreata of wild type (WT), *Nkx2.2^-/- ^*(N2.2) and *Pax6^-/- ^*(P6) mice within the total glucagon^+ ^and ghrelin^+ ^cell populations, respectively, are depicted. (M) Quantitative RT-PCR of *Arx, Pax6 *and *Nkx2.2 *mRNA in wild type, *Nkx2.2^-/-^*, *Pax6^-/- ^*and *Arx^- ^*E16.5 pancreas. (N) Quantification of Arx^+ ^cells at E16.5 in pancreata of wild type, Nkx2.2^-/- ^and *Pax6^-/- ^*mice (n = 3 pancreas per genotype). Single-factor ANOVA coupled to Newman-Keuls test was applied and p-values < 0,05 were assessed as statistically significant compared to wild type. Statistical significance is achieved with the assumption of a normal distribution. Error bars represent SEM. (original magnification: 400×, Insets magnification: 4×).

We initially focused our study at E16.5, a developmental stage subsequent to a peak in mature endocrine cell generation. In wild type pancreata, most Arx-labelled cells co-expressed glucagon and were found scattered within the pancreatic tissue (Figure 1A). Only few Arx^+^/glucagon^- ^cells were detectable, most likely corresponding to alpha-cell precursors that have not yet initiated hormone synthesis. Arx^+ ^cells did not express ghrelin, except in rare instances (Figure 1B). Interestingly, in *Nkx2.2*-deficient animals, an almost complete lack of alpha- and beta-cells accompanied by a five-fold increase in *Arx *transcript levels were noted when compared to their control counterparts ([38], Figure 1M). Subsequent quantifications revealed a 2.4-fold rise in the numbers of Arx-immunoreactive cells in E16.5 mutant pancreata compared to age-matched wild type embryos (Figure 1D, N). Clearly, this number did not fully reflect the upregulation of *Arx *transcripts, suggesting an increase in Arx production at the single cell level. These results led us to determine whether *Nkx2.2 *expression could be likewise affected in animals deficient for *Arx*. However, *Nkx2.2 *transcript levels in E16.5 *Arx *mutant pancreata were not significantly different from wild type controls, suggesting that *Nkx2.2 *transcription is not regulated by Arx (Figure 1M).

Further analyses of the Arx^+ ^cell population in *Nkx2.2*^-/- ^mice revealed that a vast majority expressed *ghrelin*. Approximately 90% of the ghrelin^+ ^cells were found positive for Arx (Figure 1E, K). Besides this ghrelin^+^/Arx^+ ^population, only few scattered Arx^+^/ghrelin^- ^or ghrelin^+^/Arx^- ^cells were detectable in this genotype. Surprisingly, most of the few remaining glucagon-expressing cells present in *Nkx2.2*-deficient pancreata were negative for *Arx *expression (Figure 1D inset, J). In order to further assess the identity of ghrelin^+^/Arx^+ ^cells, we analysed the expression of the alpha-cell-related marker gene *Brn4*. Previous analyses of *Nkx2.2 *mutant pancreata had revealed that the numbers of Brn4^+ ^cells were reduced; correlating with the lower number of glucagon^+ ^cells [23]. Consistent with these findings, the expression of Brn4 was absent from the hyperplastic ghrelin^+ ^cells noted in this genotype (Figure 1F, L). Altogether, these results suggest that, in the absence of *Nkx2.2*, the beta- and alpha-cell lineages are disfavoured and replaced by a ghrelin^+^/Arx^+^/Brn4^- ^cell fate. Considering the crucial role exerted by Arx in promoting the alpha-cell lineage, it is conceivable that such cells correspond to alpha-cell precursors that failed to acquire the full complement of alpha-cell characteristics. However, the observation that the proportion of ghrelin^+^/Arx^+^/Brn4^- ^cells present in *Nkx2.2*^-/- ^pancreata is similar to the proportion of beta-cells normally detected in wild-type mice led us to favour an alternative explanation where cells normally fated towards the beta-cell lineage fail to properly differentiate and ectopically express *Arx *and ghrelin.

The dramatic increase in *Arx *transcripts, combined with the profound loss in *Pax6 *expression in *Nkx2.2*-deficient pancreata (Figure 1M, [13,39]), prompted us to get more insights into putative interplay between these three factors. In *Pax6 *knockout mice [24,32], the early glucagon^+ ^population emerging around embryonic day 10.5 is present and properly express the associated markers *Brn4 *and *Isl1 *[25]. However, post secondary transition, glucagon^+ ^cell numbers are significantly reduced (Figure 1G, [24,25]). Consistent with these findings, it was shown that Pax6, both indirectly and directly, potentiates the expression of the *glucagon *gene [32,40,41]. Interestingly, upon *Pax6 *deficiency, the beta-cell population was found reduced concomitantly with a 5.6-fold increase in the numbers of ghrelin^+ ^cells [2,24]. Notably, both *Nkx2.2 *transcript content and expression pattern were found almost normal in Pax6^-/- ^pancreata as compared to their wild-type counterparts (Figure 1M, [25]). Surprisingly, in E16.5 *Pax6 *mutant pancreata, *Arx *transcripts were found 2.9 times increased as compared to age-matched wild-type pancreata. However, the numbers of Arx^+ ^cells were not significantly different from those observed in wild type, indicating that the deficiency in *Nkx2.2 *or *Pax6 *leads to a similar increase in *Arx *transcripts at the single cell level (Figure 1M, N). Interestingly, and similar to what we observed in *Nkx2.2-*deficient mice, most of the Arx^+ ^cells present in *Pax6*-deficient pancreata, express ghrelin (Figure 1H, inset). Approximately 80% of the ghrelin^+ ^cells were found to express Arx, although scattered Arx^+^/ghrelin^- ^and ghrelin^+^/Arx^- ^cells were also detectable (Figure 1K). The expression of Brn4 appeared unchanged in *Pax6*-deficient mice at E14.5 and E16.5 ([25] and Figure 1I, respectively). Brn4 expression was clearly noted in approximately 45% of the ghrelin-expressing cells, suggesting at least a partially overlapping expression with *Arx *(Figure 1L). Notably, beside the ghrelin^+^/Brn4^+ ^cell population, Brn4^+ ^cells were also found outside of the ghrelin-stained areas (Figure 1I). Together, our data indicate that the reduction of alpha- and beta-cell numbers in *Nkx2.2^-/- ^*or *Pax6^-/- ^*pancreata is accompanied by an increase in the numbers of cells co-expressing Arx and ghrelin. Interestingly, the loss of *Arx *does not seem to impact on Nkx2.2 or Pax6 expression.

### Comparative analysis of ghrelin^+ ^cells in pancreata of wild type, *Nkx2.2*^-/- ^and *Pax6*^-/- ^mice

**Figure 2:**
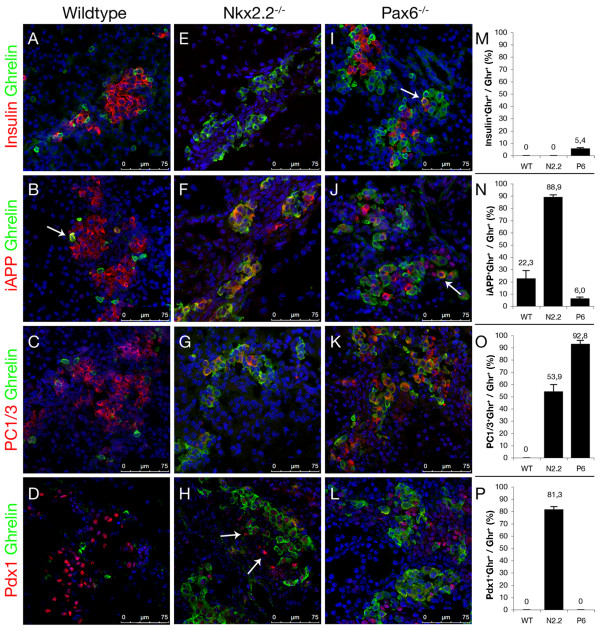
**Co-detection of ghrelin and insulin, iAPP, PC1/3 or Pdx1 in pancreata of wild type, *Nkx2.2^-/- ^*and *Pax6^-/- ^*mice at E17.5**. (A-D) Wild type ghrelin^+ ^cells do not express insulin, PC1/3 or Pdx1. However, scattered ghrelin^+ ^cells express iAPP (Arrow). (E-H) No insulin reactive cells are detectable in *Nkx2.2*-deficient pancreata. The majority of ghrelin^+ ^cells express iAPP and low levels of Pdx1. Scattered iAPP^+^/ghrelin^- ^(F) and Pdx1^+^/ghrelin^- ^cells (H, Arrows) are detectable. (G) PC1/3 is associated with a subpopulation of ghrelin-expressing cells in *Nkx2.2*-deficient pancreata. (I-L) In pancreata deficient for *Pax6 *the population of ghrelin^+ ^cells does not express insulin or iAPP except in very rare instances (Arrows in I+J). Almost all *Pax6*-deficient ghrelin^+ ^cells express PC1/3 but none produce Pdx1. Counterstaining with DAPI. (M-P) The percentages of insulin^+^/ghrelin^+^, iAPP^+^/ghrelin^+^, PC1/3^+^/ghrelin^+ ^and Pdx1^+^/ghrelin^+ ^cells in wild type (WT), *Nkx2.2^-/- ^*(N2.2) and *Pax6^-/- ^*(P6) mice within the total ghrelin^+ ^cell population are depicted. Pictures were taken using confocal microscopy.

To further assess the identity of the ghrelin^+^/Arx^+ ^cell population arising in pancreata of *Nkx2.2*^-/- ^and *Pax6*^-/- ^mice, we assayed the expression of a number of beta-cell-related markers, such as insulin, iAPP, PC1/3 and Pdx1 at E17.5 (Figure 2). Most of these proteins have previously been found expressed in both mutant pancreata, however their expression relative to ghrelin has not been fully resolved [2,13,42].

Previous detailed analyses of wild type ghrelin-expressing cells had led to the conclusion that these could be subdivided into glucagon^+^ghrelin^+ ^alpha-cells and glucagon^-^ghrelin^+ ^epsilon-cells. It should be noted that only a subset of alpha-cells were actually found positive for ghrelin, such cells retaining glucagon, Arx and Brn4 expression (Figure 1B, C, [2,13]). For technical reasons, we did not distinguish between these two populations in our analysis of wild type pancreata. Notably, none of the wild type ghrelin^+ ^cells did express insulin, PC1/3, or Pdx1 (Figure 2A, C, D). However, approximately 20% of ghrelin^+ ^cells expressed iAPP at E17.5 (Figure 2B Arrow, N).

Next, we analysed the distribution of these markers within the hyperplastic ghrelin^+ ^population found in *Nkx2.2*-deficient pancreata. Notably, Pdx1 expression had been detected in these cells, although at markedly lower levels compared to wild type pancreata [22]. Our analysis revealed, that approximately 80% of ghrelin-expressing cells expressed low levels of Pdx1 (Pdx1^low^) and iAPP in this genotype (Figure 2H, P). Moreover, we frequently detected Pdx1^+^/ghrelin^- ^cells close to hormone-producing cells in the pancreatic epithelium of such animals (Figure 2H, Arrows). The prohormone convertase 1/3 was found localized in approximately 54% of ghrelin^+ ^cells (Figure 2G, O). Thus, the majority of the ghrelin^+^/Arx^+ ^cells that emerge upon *Nkx2.2 *deficiency significantly differ from wild type ghrelin^+ ^cells. The direct association of these beta-cell markers with ghrelin^+^, and hence with Arx^+ ^cells, provides a strong evidence that at least a subpopulation of the ghrelin^+^/Arx^+ ^cells exhibit committed beta-cell characteristics, and do not consist of putative alpha-cell precursors.

Interestingly, similar analyses of the ghrelin^+^/Arx^+ ^population in *Pax6*-deficient pancreata outlined different alterations. Notably, a few apparently normal beta-cells develop in the absence of *Pax6*, these expressing insulin, Pdx1, Hb9, Nkx6.1 and MafA [2,42]. However, we did not detect co-expression of ghrelin and insulin, except in very rare instances (Figure 2I Arrow, M). At comparably low frequencies were detected iAPP^+^/ghrelin^+ ^co-expressing cells, however, the bulk of ghrelin^+ ^cells appeared negative for iAPP (Figure 2J, N). Moreover, and in contrast to the ghrelin^+ ^population noted in *Nkx2.2*-deficient pancreata, we did not observe any co-expression of ghrelin and Pdx1 in pancreata of such mice (Figure 2L, [2]). Surprisingly, almost all ghrelin^+ ^cells were immunoreactive for PC1/3 (Figure 2K, O). Consequently, our data reveal significant differences in the expression profile of ghrelin^+ ^cells arising in pancreata of *Nkx2.2*^-/- ^and *Pax6*^-/- ^mice. While most ghrelin^+ ^cells from *Nkx2.2*-deficient embryos are positive for iAPP and Pdx1^low^, the majority of the *Pax6*^-/- ^ghrelin-cells do not express these markers. Importantly, the expression of *iAPP *and *Pdx1 *is not *per se *impeded upon *Pax6 *deficiency as it can clearly be detected outside of the ghrelin-stained areas, probably labelling the remaining beta-cells. Hence, our data supports the notion that, upon the loss of *Nkx2.2 *or *Pax6 *functional alleles, endocrine precursor cells are still committed towards the beta-cell lineage, do initiate the expression of some beta-cell-specific determinants, but fail to reach a mature beta-cell state and rather misexpress *Arx *and *ghrelin*.

**Figure 3:**
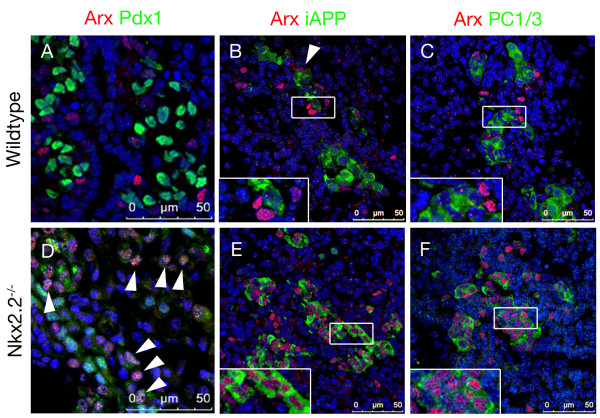
**Co-detection of Arx and Pdx1, iAPP or PC1/3 in pancreata of wild type and *Nkx2.2^-/- ^*mice at E17.5**. (A-C) Wild type Arx^+ ^cells do not express Pdx1 (A) or PC1/3 (C, inset). Most Arx^+ ^cells do not express iAPP (B), except in rare instances (Arrowhead). (D-F) The majority of Arx^+ ^cells in Nkx2.2^-/- ^pancreata express Pdx1^low ^(Arrows), iAPP (E, inset) and PC1/3 (F, inset). Counterstaining with DAPI. Pictures were taken using confocal microscopy (Insets magnification: 4×).

Next we assayed the expression of Pdx1, iAPP and PC1/3 in relation with Arx in *Nkx2.2^-/- ^*pancreata. Arx^+ ^cells do not express Pdx1 or PC1/3 in wild type pancreata at E17.5 (Figure 3A, C). Scattered Arx^+ ^cells co-express iAPP at this age; however the bulk does not (Figure 3B, inset). In contrast, the majority of the Arx^+ ^cells arising in *Nkx2.2^-/- ^*pancreata do co-express Pdx1^low^, iAPP or PC1/3 (Figure 3D, Arrows, E, F).

### *Nkx2.2^-/-^Arx^- ^*mice pancreata lack alpha- and mature beta-cells and exhibit a hyperplastic ghrelin/somatostatin co-positive cell population

Our results pointing towards a putative failure of beta-cell development upon *Nkx2.2 *deficiency, prompted us to investigate the role of Arx in this context. Specifically, we asked (1) whether the loss of *Nkx2.2 *is directly responsible for this failure or (2) whether the subsequent increase in *Arx *expression, resulting from *Nkx2.2 *deficiency, could lead to such phenotypic alteration. To address this issue and gain further insight into the mutual roles of Nkx2.2 and Arx in the processes underlying the specification towards the alpha- and beta-cell lineages, we generated animals doubly mutant for *Nkx2.2 *and *Arx*.

**Figure 4:**
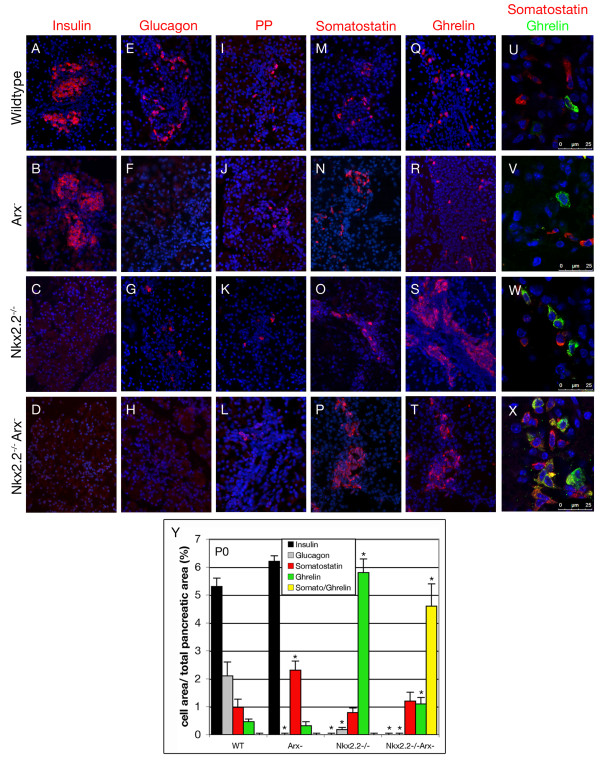
**Expression of endocrine hormones in pancreata of wild type, *Arx*^-^, *Nkx2.2*^-/- ^and *Nkx2.2^-/-^Arx^- ^*newborns**. Insulin (A-D), glucagon (E-H), pancreatic polypeptide (I-L), somatostatin (M-P) and ghrelin (Q-T) immunofluorescence-detection at P0. (D, H) Note that both beta- and alpha-cells lack in *Nkx2.2^-/-^Arx^- ^*double-mutant mice (original magnification: 200×). (U-X) Co-detection of ghrelin and somatostatin in P0 pancreata of wild type, *Arx*^-^, *Nkx2.2*^-/- ^and *Nkx2.2^-/-^Arx^- ^*mice. Somatostatin^+ ^cells and ghrelin^+ ^cells label distinct cell populations in wild type (U), *Arx*- (V) and *Nkx2.2*-deficient (W) pancreata. (X) However, in *Nkx2.2^-/-^Arx^- ^*double mutant mice a population of ghrelin^+^/somatostatin^+ ^co-positive cells is detectable besides single-hormone-expressing ghrelin^+ ^or somatostatin^+ ^cells. Pictures in U-X were taken using confocal microscopy. Counterstaining with DAPI. (Y) Quantification of hormone producing cells (without PP-cells) in pancreata of wild type, *Arx*^-^, *Nkx2.2*^-/- ^and *Nkx2.2^-/-^Arx^- ^*mice at P0. Bars represent percentage of fluorescent cell area per total pancreatic area. n = 3 pancreas per genotype. Single-factor ANOVA coupled to Newman-Keuls test was applied and p-values < 0,05 were assessed as statistically significant compared to wild type. Statistical significance is achieved with the assumption of a normal distribution. Error bars represent SEM.

Double mutant mice were born following normal Mendelian inheritance, indicating no embryonic lethality. In contrast to their *Nkx2.2*- and *Arx*-single mutant littermates surviving until postnatal day (P) 5 and 2, respectively, compound animals did not suckle and died within few hours *postpartum*. Analyses of their blood glucose levels indicated a hypoglycaemic condition with blood glucose levels lower than 20 mg/dl. Using immunofluorescence, we examined the different endocrine cell populations at P0 (Figure 4). Although *Arx*-single mutants were found to exhibit a slight increase in the number of beta-cells, we could not detect any insulin-immunoreactive cells in compound mutant pancreata, further demonstrating that functional *Nkx2.2 *is essential for the formation of beta-cells (Figure 4A-D, [27]). As reported in *Arx*-deficient pancreata, glucagon-immunoreactive cells were lost in double-mutant mice (Figure 4F, H), concurring with previous findings outlining Arx as an alpha-cell lineage-defining factor [27,36,37]. PP-expressing cells were detected in wild type, single- and double-mutant mice although relatively rare in pancreata of *Nkx2.2^-/- ^*and *Nkx2.2^-/-^Arx^- ^*newborns (Figure 4I-L).

Next, we assayed somatostatin-expressing delta-cells. Wild type delta-cells classically do not express *Nkx2.2*. Accordingly, they appeared in normal numbers in *Nkx2.2*-deficient mice (Figure 4O, [22,23]). As previously reported, the delta-cell population was found significantly increased in *Arx*-mutants as compared to age-matched controls (2.4-fold) (Figure 4N, [27]). Hence, we expected an unopposed delta-cell differentiation in the absence of both factors. Importantly, we noticed a dramatic increase in the number of somatostatin-producing cells at all stages tested (E14.5, E18.5 and P0) as compared to wild type, *Arx*- or *Nkx2.2*-deficent pancreata (Figure 4M-P, data not shown).

In P0 wild type pancreata, a small population of single-hormone ghrelin^+ ^epsilon-cells is detectable beside glucagon^+^/ghrelin^+ ^alpha-cells [2]. The loss of functional *Arx *specifically depletes the population of glucagon^+^/ghrelin^+ ^alpha-cells but does not significantly impact on the numbers of single-hormone ghrelin^+ ^epsilon-cells [2,27]. Similarly, glucagon^+^/ghrelin^+ ^alpha-cells were found lacking in pancreata of Nkx2.2^-/- ^animals [13]. However, in the absence of both *Nkx2.2 *and *Arx*, a drastic augmentation in ghrelin^+ ^cells was observed, such increase being similar to that of *Nkx2.2*^-/- ^pancreata (Figure 4S, T). Surprisingly, somatostatin and ghrelin co-detection revealed a great number of cells co-expressing both hormones (Figure 4X, Y). This later result was confirmed using two different sets of anti-ghrelin and anti-somatostatin antibodies (Figure 4 and data not shown). Notably, such bi-hormonal cells were not detectable in P0 wild type, *Nkx2.2*- or *Arx *single mutant mice (Figure 4U-W). Moreover, pancreata of double heterozygous mice as well as of *Nkx2.2*^-/-^*Arx*^+/- ^or *Nkx2.2*^+/-^*Arx*^- ^mice did not display such bi-hormonal cells (data not shown).

We further quantified the different endocrine cell subtypes present at P0 (Figure 4Y). In *Nkx2.2*^-/- ^pancreata, the numbers of ghrelin^+ ^cells was found approximately 12 times increased compared to ghrelin^+ ^cells in wild type pancreata. Interestingly, a similar augmentation in the total amount of ghrelin^+ ^cells was noted comparing pancreata of *Nkx2.2 *single and *Nkx2.2/Arx *double mutants; however, approximately 80% of such ghrelin^+ ^cells also expressed the somatostatin hormone in the latter genotype. This suggests that Arx suppresses somatostatin expression in Nkx2.2^-/- ^ghrelin^+ ^cells. It should be noted that the numbers of single-hormone positive somatostatin-producing cells observed in *Nkx2.2*^-/-^*Arx*^- ^were comparable to those of *Nkx2.2*^-/- ^single knockout and wild type pancreata. Equally important is the observation that the content of ghrelin^+^somatostatin^+ ^cells found in *Nkx2.2*^-/-^*Arx*^- ^or of ghrelin^+ ^noted in *Nkx2.2*^-/- ^were similar to the beta-cell numbers found in their wild type counterparts. This finding further supports the notion that beta-cells are correctly specified but fail to properly differentiate in these genotypes. Altogether, our findings demonstrate that the combined loss of *Nkx2.2 *and *Arx *prevents the formation of both insulin- and glucagon-expressing endocrine cells, but has no effect on the number of single-hormone positive somatostatin-producing cells. However, an alternative cell subtype develops, such cells expressing both ghrelin and somatostatin hormones.

### *Nkx2.2/Arx-*deficient endocrine cells express *iAPP *as well as low levels of *Pdx1 *and *Pax6*

**Figure 5:**
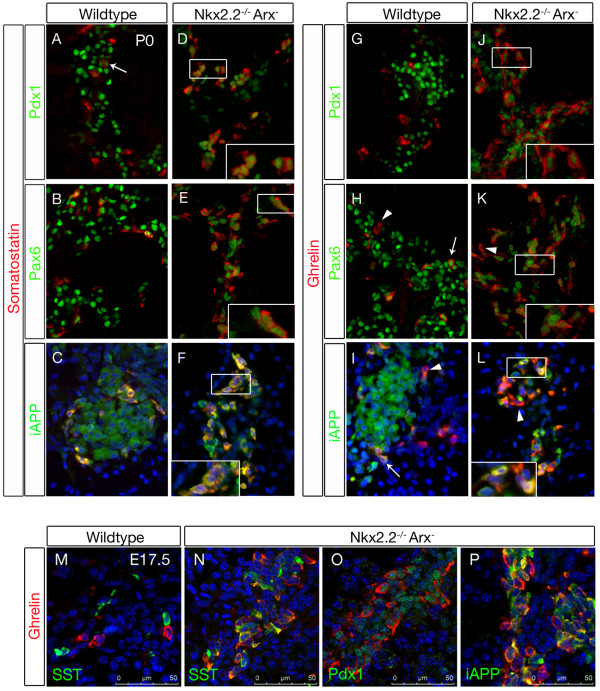
**Expression of *Pdx1*, *Pax6 *and *iAPP *in pancreata of wild type and *Nkx2.2^-/-^Arx^- ^*newborns**. (A) In wild type pancreata, a subpopulation of delta-cells expresses low levels of Pdx1 (Arrow) while the bulk does not. (B, C) Wild type delta-cells express Pax6 and iAPP. (D, inset) Notably, in *Nkx2.2^-/-^Arx^- ^*mice almost all somatostatin^+ ^cells display low levels of Pdx1. (E, F, insets) *Nkx2.2^-/-^Arx^- ^*somatostatin^+ ^cells are immunoreactive for iAPP and low levels of Pax6. (G) Wild type epsilon-cells do not express Pdx1. (H, I) Pax6 or iAPP can be detected in a subset of wild type ghrelin^+ ^cells (Arrows); a few ghrelin^+ ^cells are negative for Pax6 or iAPP (Arrowheads). (J, K) In *Nkx2.2^-/-^Arx^- ^*pancreata, the majority of ghrelin^+ ^cells are found positive for low levels of Pdx1 and Pax6; scattered ghrelin^+ ^cells do not express Pax6 (K, Arrowhead). (L, inset) Islet amyloid polypeptide is detectable in the majority of ghrelin^+ ^cells; some are negative for iAPP (Arrowhead). Pictures in D, E, J and K have been captured using a 2-fold exposure time compared to respective control images in order to allow for a clear detection of Pdx1 and Pax6 (original magnification 800×). C, F, I and L are counterstained with DAPI. (M-N) Confocal images of E17.5 wild type and *Nkx2.2/Arx *double-mutant pancreata. Similar to the expression patterns in newborns, *Nkx2.2/Arx *double-deficient ghrelin^+ ^cells express somatostatin, Pdx1^low ^and iAPP at E17.5.

*Pdx1 *expression persists in developing and mature wild type beta-cells and can be found in scattered delta-cells, these two endocrine cell subtypes also expressing islet amyloid polypeptide (IAPP) ([17,43-45] and Figure 5C). Our observations prompted us to further characterize the identity of the endocrine cells present in the pancreata of *Nkx2.2^-/-^Arx^- ^*mice by investigating the expression of Pdx1 and iAPP in association with somatostatin or ghrelin. Moreover, we also assessed the expression of the endocrine marker Pax6 that had been suggested to be Nkx2.2-dependent [13].

In contrast to the scattered expression of Pdx1 classically observed in delta-cells of wild type, *Nkx2.2^-/- ^*or *Arx^- ^*embryos, Pdx1 could be detected in almost all somatostatin^+ ^cells present in *Nkx2.2^-/-^Arx^- ^*double mutant pancreata. Notably, Pdx1 expression levels were markedly lower as compared to wild type pancreata but comparable to the low Pdx1 levels found in *Nkx2.2*^-/- ^pancreata (Figure 5D, [39]). Furthermore, all somatostatin^+ ^cells were found to express *iAPP *(Figure 5F) and low levels of *Pax6 *(Figure 5E). Subsequent analyses of ghrelin-secreting cells in double mutant pancreata expectedly revealed that a vast majority likewise expressed these factors (Figure 5J-L). The expression of these beta-cell associated genes can also be observed in double-mutant pancreata at embryonic day 17 (Figure 5O, P). Hence, the alterations of Pdx1, iAPP and Pax6 expression appear similar in mice lacking *Nkx2.2 *and animals deficient for both *Nkx2.2 *and *Arx*, outlining the importance of Nkx2.2 in sustaining normal expression levels of these genes. Consequently, the main endocrine cell type found in pancreata of double mutant mice is characterized by the expression of ghrelin, somatostatin, Pdx1^low^, Pax6^low ^and iAPP. Therefore, such cells display the same beta-cell-like features as the ghrelin^+ ^cells found in *Nkx2.2 *single mutants and differ only in somatostatin expression. Taken into account that, in *Nkx2*.2 single mutants, the population of ghrelin^+^/Pdx1^low^/iAPP^+ ^cells is also expressing high levels of *Arx*, it is conceivable that the loss of Arx promotes ectopic somatostatin expression in such cells.

### Expression of *Pax4 *and *Nkx6.1 *in pancreata of normal, single- and double-mutant animals

**Figure 6:**
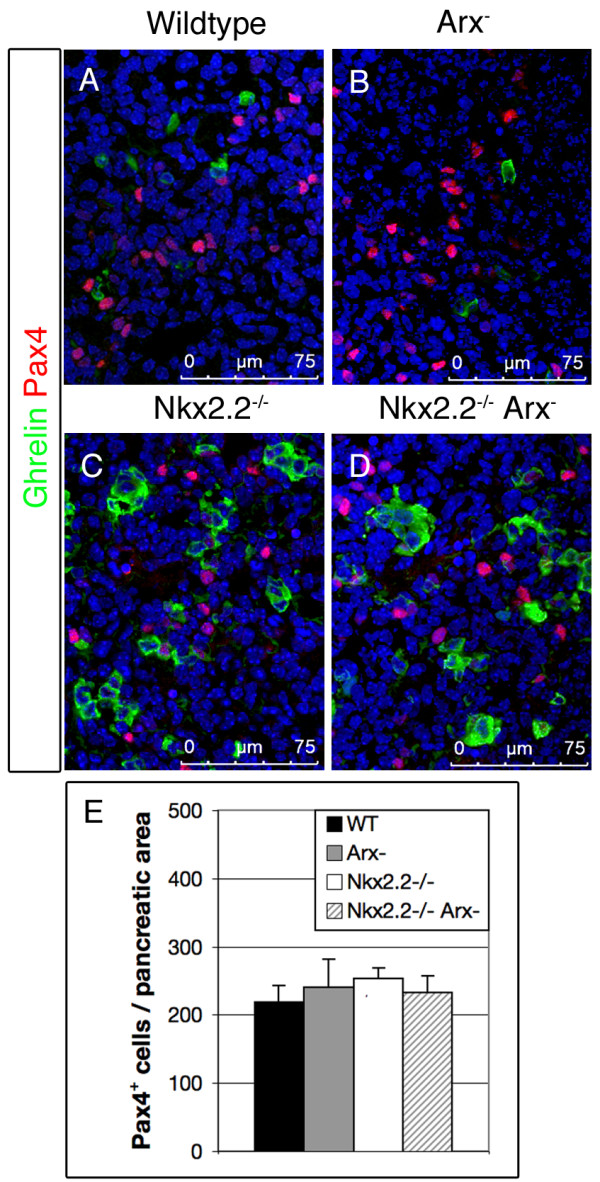
***Pax4 *expression in pancreata of wild type, *Arx^-^*, *Nkx2.2^-/- ^*and *Nkx2.2^-/-^/Arx^- ^*at E16.5**. (A-D) Pax4-expressing cells are present in normal numbers at E16.5 in pancreata of wild type, *Arx^-^*, *Nkx2.2^-/- ^*and *Nkx2.2^-/-^/Arx^- ^*mice. Notably, no co-expression of Pax4 and ghrelin is detectable in wild type or mutant pancreata. Pictures were taken using confocal microscopy. (E) Quantification of Pax4^+ ^cells at E16.5 in pancreata of wild type, *Arx^-^*, *Nkx2.2^-/- ^*and *Nkx2.2^-/-^Arx^- ^*mice (n = 3 pancreas per genotype). Single-factor ANOVA coupled to Newman-Keuls test was applied and p-values < 0,05 were assessed as statistically significant compared to wild type. Statistical significance is achieved with the assumption of a normal distribution. Error bars represent SEM.

To further determine the developmental stage at which beta-cell differentiation is impaired in our mutant animals, we subsequently assayed the expression of the endocrine precursor marker Pax4 (Figure 6, [26,34]). Broadly expressed in the embryonic pancreas around E16.5, its expression successively declines and is rarely detectable in newborns [39]. However, low expression levels have been noted in adult beta-cells [35]. Moreover, Pax4 has been shown to be essential for the formation of beta- and delta-cells, and for the expression of a normal set of genes associated with beta-cell characteristics. In accordance with previous studies detecting *Pax4 *transcripts in *Arx*- or *Nkx2.2*-deficient mice, we observed numerous Pax4^+ ^cells at embryonic day 16.5 in the pancreatic epithelium of such animals using immunohistochemical detection (Figure 6B, C). We have previously reported that the Arx factor binds the *Pax4 *promoter and negatively regulates *Pax4 *expression, thereby specifying alpha vs. beta-cell fates [36,37]. However, despite markedly elevated *Arx *transcript levels upon the loss of functional *Nkx2.2*, we did detect normal Pax4^+ ^cell numbers in pancreata of E16.5 *Nkx2.2^-/- ^*mice, suggesting that, in this genotype and at this age, Pax4 is not downregulated by Arx. Interestingly, the combined loss of *Nkx2.2 *and *Arx *did not impact in the number of Pax4^+ ^cells as compared to *Nkx2.2 *mutant pancreata, thus indicating that their formation does not depend on either factor (Figure 6D, E). Notably, and in agreement with previous findings, Pax4 expression was not detectable in ghrelin^+ ^cells of wild type or mutant pancreata.

**Figure 7:**
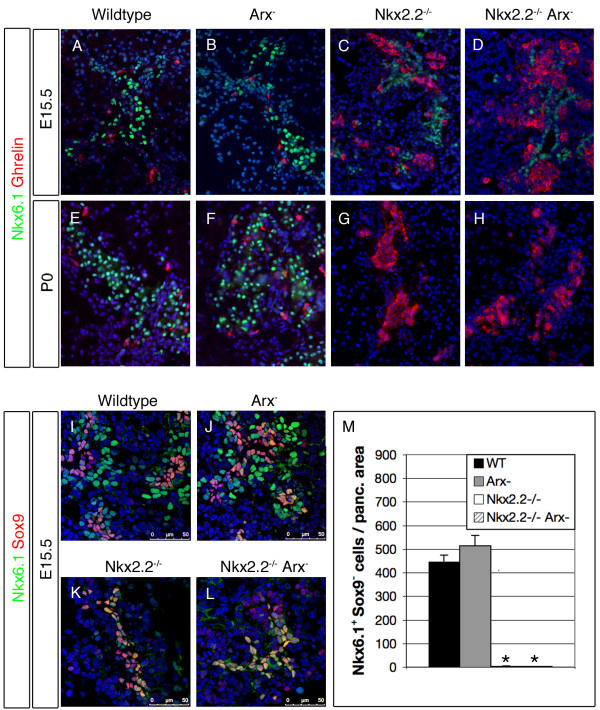
***Nkx6.1 *expression is differentially dependent on Nkx2.2**. (A-D) Nkx6.1 is broadly expressed in the pancreatic epithelium of wild type, *Arx^-^*, *Nkx2.2^-/- ^*and *Nkx2.2^-/-^Arx^- ^*mice at embryonic day 15.5. (E, F) In wild type and *Arx*-mutants, Nkx6.1 expression is maintained and detectable at P0. (G, H) However, in the absence of *Nkx2.2 *or *Nkx2.2/Arx*, a steady decrease in Nkx6.1^+ ^cell numbers is detected from E15.5 on so that Nkx6.1^+ ^cells are not detectable in pancreata of newborns. No co-expression of Nkx6.1 and ghrelin is detectable in wild type or mutant tissues at the investigated developmental time-points (original magnification 400×). (I-L) Nkx6.1^+^Sox9^+ ^ductal/endocrine progenitors are present in all four genotypes at E15.5. In wild type and *Arx*-mutants Nkx6.1^+^Sox9^- ^cells are detected in comparable numbers. (K, L) However, in the absence of Nkx2.2 or Nkx2.2 and Arx no such cells are detectable. Pictures in I-L were taken using confocal microscopy. (M) Quantification of Nkx6.1^+^Sox9^- ^cell numbers in E15.5 pancreata of wild type, *Arx^-^*, *Nkx2.2^-/- ^*and *Nkx2.2^-/-^Arx^- ^*mice (n = 3 pancreas per genotype). Single-factor ANOVA coupled to Newman-Keuls test was applied and p-values < 0,05 were assessed as statistically significant compared to wild type. Statistical significance is achieved with the assumption of a normal distribution. Error bars represent SEM.

Next, we analysed the temporal expression of Nkx6.1 in pancreata of all four genotypes (Figure 7). Importantly, previous studies had shown that both Pax4 and Nkx2.2 are essential to maintain the expression of Nkx6.1 in developing beta-cells [23,39]; in the absence of either factor, a decrease in Nkx6.1^+ ^cell numbers was seen from E16.5 on, progressing to a complete loss of Nkx6.1^+ ^cells in newborns. The loss of *Arx *had no impact on Nkx6.1 expression as it was found maintained and properly located in emerging beta-cells (Figure 7F, [27]). In *Nkx2.2^-/-^Arx^- ^*compound mutant mice, we observed a temporal expression pattern of Nkx6.1 comparable to that of *Nkx2.2*-deficient pancreata; broadly expressed in the undifferentiated pancreatic epithelium at E15.5, the number of Nkx6.1^+ ^cells was found drastically reduced at E18.5, while no Nkx6.1^+ ^cells could be detected at P0 (Figure 7C, D, G, H and data not shown).

Recent studies have demonstrated that Nkx6.1^+ ^cells arising during embryonic pancreatogenesis can be subdivided into Sox9^+ ^and Sox9^- ^cells, representing ductal/endocrine progenitors and committed beta-cells, respectively [31,46,47]. In order to characterize the Nkx6.1^+ ^population within the different genotypes, we co-detected Nkx6.1 and Sox9 using immunofluorescence. In E15.5 wild type and *Arx*-mutant pancreata, numerous Nkx6.1^+^Sox9^- ^beta-cells are detectable besides Nkx6.1^+^Sox9^+ ^progenitor cells (Figure 7I, J, M). Interestingly, in *Nkx2.2*-deficient genotypes, Nkx6.1^+ ^cells were found associated to the uncommitted progenitor population (Figure 7K, L, M). These data clearly demonstrate that the expression of *Nkx6.1 *is not dependent on Nkx2.2 activities within Sox9^+ ^progenitor cells. However, as soon as a cell enters the endocrine lineage, *Nkx6.1 *expression is found highly dependent on Nkx2.2. These results also imply that the steady decline in Nkx6.1^+ ^cell numbers observed in Nkx2.2^-/- ^pancreata, most likely reflects the normal decrease of progenitor cells classically noted in the last days of embryonic pancreas development [47].

Taken together, our results demonstrate that *Nkx2.2 *and *Arx *are dispensable for the generation of Pax4^+ ^cells. Moreover, the alterations in the expression of Pdx1, Nkx6.1 and Pax6, appear similar when comparing pancreata of *Nkx2.2*-single and *Nkx2.2/Arx *compound-mutants, suggesting that the failure of beta-cell differentiation and the associated *ghrelin *misexpression are solely caused by the loss of *Nkx2.2*. Similarly, the dramatic alterations in alpha-cell development in *Nkx2.2*^-/- ^pancreata, where *Arx *expression is even more pronounced, sustain the notion that *Nkx2.2 *is also necessary for the acquisition of mature alpha-cell fate.

## Discussion

In this study we have (re-) examined the consequences of the loss-of-function of *Pax6*, *Arx *or *Nkx2.2*, as well as of the combined loss of *Nkx2.2 *and *Arx*, on the development of pancreatic endocrine hormone-producing cells. Previous studies had shown that the loss of *Nkx2.2 *or *Pax6 *results in the formation of numerous ghrelin-expressing cells at the expense of the beta- and alpha-cell populations [2,13]. Herein, we demonstrate that the majority of such ghrelin^+ ^cells express the alpha-cell specifier Arx. However, further expression analyses focusing on ghrelin and alpha- or beta-cell-related genes suggest a partial association of the mutant ghrelin^+^/Arx^+ ^population to the beta-cell lineage. The combined loss of *Nkx2.2*- and *Arx *likewise results in the formation of numerous ghrelin^+ ^cells at the expense of alpha- and beta- cells, whereas delta-cell formation appears unaffected. Notably, the majority of the ghrelin^+ ^cells were, yet again, found to display some of the features classically associated to beta-cells, but also to ectopically express somatostatin, suggesting that the additional loss of *Arx *promotes the ectopic expression of *somatostatin*.

### Ghrelin^+ ^cells arising in *Nkx2.2*-deficient pancreata display different characteristics as compared to ghrelin^+ ^cells arising in wild type or *Pax6*-deficient mice

Previous reports have revealed that the loss of *Pax4*, *Nkx2.2 *or *Pax6 *is accompanied by the emergence of numerous ghrelin^+ ^cells at the expense of the beta-cell population [2,13,33]. Notably, in either mutant pancreas, the expression of the two other genes is detectable, rendering it difficult to attribute putative *ghrelin*-suppressing activities to one of the three candidates. At least for Pax4, such *ghrelin*-suppressing function has been demonstrated *in vitro*, whereas Nkx2.2 has been shown to exhibit *ghrelin*-activating functions in cell culture experiments [33,48]. However, *Pax4 *expression remains detectable in *Nkx2.2*- or *Pax6*-mutant pancreata, suggesting that *ghrelin*-silencing requires multiple factors. Interestingly, in all three mutants, *Arx *transcripts levels are found increased and Arx is often associated with ghrelin-expressing cell populations ([33,38], this study). The possibility that Arx acts as a putative *ghrelin*-activator, however, appears unlikely as *ghrelin*-expression is also broadly maintained in cells arising in *Nkx2.2/Arx *compound mutants.

In an effort to better characterize such mutant ghrelin-expressing cells, we analysed co-expression of ghrelin and alpha-or beta-cell related markers in pancreata of wild type, *Pax6^-/- ^*and *Nkx2.2^-/- ^*mice. The bulk of the *Nkx2.2*-deficient ghrelin^+ ^cells expresses Arx, iAPP, PC1/3 and Pdx1 and thus strongly differs from wild type epsilon-cells, excluding a specific augmentation of epsilon-cell numbers upon *Nkx2.2 *depletion. As previously suggested, our data strengthens the notion, that the majority of ghrelin^+ ^cells arising in *Nkx2.2*-deficient mice represent committed beta-cells that fail to properly differentiate [22,23].

Wang et al. suggested that Nkx2.2 acts in synergy with Pax4 during the early steps of beta-cell differentiation [39]. Interestingly, the population of ghrelin^+^/glucagon^+ ^cells emerging in pancreata of *Pax4*-deficient mice share many characteristics with the one observed in *Nkx2.2*-deficient mice, including the expression of Arx, iAPP, Pdx1^low ^and PC1/3 ([33], data not shown). It is therefore conceivable, that such cells likewise represent committed beta-cell precursors and that the presence of Nkx2.2 and Arx allows their maturation towards a glucagon-expressing state. Wang et al. also outlined that synergistic activities of Nkx2.2 and Pax4 are essential in the early steps of beta-cell differentiation in order to activate crucial factors, such as Hb9, Nkx6.1 and Pdx1^high ^[39]. Furthermore, we previously demonstrated that Pax4 strongly suppresses *Arx *expression in early beta-cells [27,36]. Together with these findings, our current data provides strong evidence for the essential synergistic activity of Nkx2.2 and Pax4 to suppress *Arx *expression in early committed beta-cells. A strict control of Arx production appears essential as Arx is sufficient to convert mature beta-cells into PP-like or alpha-like cells [37]. The facts that Nkx2.2 is also expressed in alpha-cells and that alpha-cell development is impaired upon *Nkx2.2 *deficiency, lead to the assumption that the lineage determining activities of both Arx and Pax4 require Nkx2.2 activity.

We also demonstrate that the lack of functional *Pax6 *results in the formation of ghrelin^+^/Arx^+ ^cells. Pax6 was thought to exert a more general role in the maintenance of the beta-cell identity. The presence of a few but apparently normal beta-cells in pancreata of *Pax6*-deficient mice even led to the suggestion of an alternate Pax6-independent pathway leading to mature beta-cells [42]. Interestingly, we frequently detected scattered insulin^+^/ghrelin^+ ^cells in such mutant pancreata. Such cells might represent a transitional state of beta-cells converting into ghrelin^+ ^cells. Tracing the lineage of insulin-expressing cells in the *Pax6*-deficient background would doubtless shed more light on this interesting issue. However, *Pax6*-deficient ghrelin^+ ^cells express neither iAPP nor Pdx1, thus differing from the ghrelin^+ ^populations arising in *Nkx2.2*- or *Pax4*-deficient pancreata. The presence of iAPP and Pdx1 in the remaining *Pax6*-deficient beta-cells indicates that *Pax6 *is not necessary for the activation of these genes. However, it is conceivable that Pax6 function is essential for maintaining their expression as soon as a beta-cell reaches a certain stage of differentiation.

The almost complete loss of glucagon^+ ^cells in *Pax6*-deficient mice indicates a more essential role of *Pax6 *in alpha-cells. In this context, it has been demonstrated that Pax6 binds and activates the *glucagon *gene [32,40,41,49], however it remains unknown whether Pax6 is also involved in the determination of the alpha-cell lineage. The continued expression of three alpha-cell related genes (*Nkx2.2, Arx, Brn4*) in *Pax6*-deficient pancreata might be an indication for a predominant involvement of *Pax6 *in later stages of alpha-cell differentiation.

### Ghrelin^+^/somatostatin^+ ^cells arising in *Nkx2.2/Arx *compound mutants represent a non-delta-cell type

Our analysis shows that the combined loss of *Nkx2.2 *and *Arx *prevents the formation of mature alpha- and beta-cells, thus confirming the requirement of Arx and Nkx2.2 activities for the formation of these cell types. However, the presence of Pax4^+ ^cells indicates that an endocrine precursor state is attained without *Nkx2.2 *and/or *Arx*. This, and previous studies, suggest that *Nkx2.2 *is essential for the maintenance of high levels of Pdx1 and Pax6 as well as the activation of *Nkx6.1 *in developing beta-cells [13,22,39]. Consistent with these assumptions, the alterations found in the expression of Pdx1, Pax6 and Nkx6.1 appear similar, comparing pancreata of *Nkx2.2 *single- and *Nkx2.2/Arx *compound-mutant mice. These data once more imply that *Arx *is dispensable for the beta-cell fate and that the proper expression of these genes can be attributed to *Nkx2.2 *alone.

However, the additional loss of *Arx *results in the formation of numerous ghrelin^+^/somatostatin^+ ^cells beside populations of single-hormone positive somatostatin^+ ^or ghrelin^+ ^cells. We did not detect *ghrelin *expression in delta-cells arising in wild type or either single-knockout pancreata. Together with the observation that neither Nkx2.2 nor Arx is expressed in wild type delta-cells, we conclude that such double positive cells do not represent delta-cells ectopically expressing ghrelin but rather a population comparable to the ghrelin^+ ^cells arising in *Nkx2.2*-deficient mice. This is further supported by the fact that ghrelin^+^/somatostatin^+ ^cells display the same broad expression of Pdx1^low ^as observed in *Nkx2.2*-deficient ghrelin^+ ^cells. Hence, we conclude that 80% of the ghrelin^+ ^cells initiate somatostatin expression upon the additional loss of *Arx*. Not all ghrelin-labelled cells co-express somatostatin in double-mutant pancreata. This might be attributed to their distinct predeterminations and partially reflect the presence of normal epsilon-cells. Nevertheless, our data also imply that *Arx *or a not yet identified downstream mediator exerts a suppressive effect on *somatostatin *expression. In this context, previous studies demonstrated that Arx activities strongly antagonize delta-cell formation [27,36,37]. However, whether Arx directly antagonizes *somatostatin *expression would require further investigations.

## Conclusions

**Figure 8:**
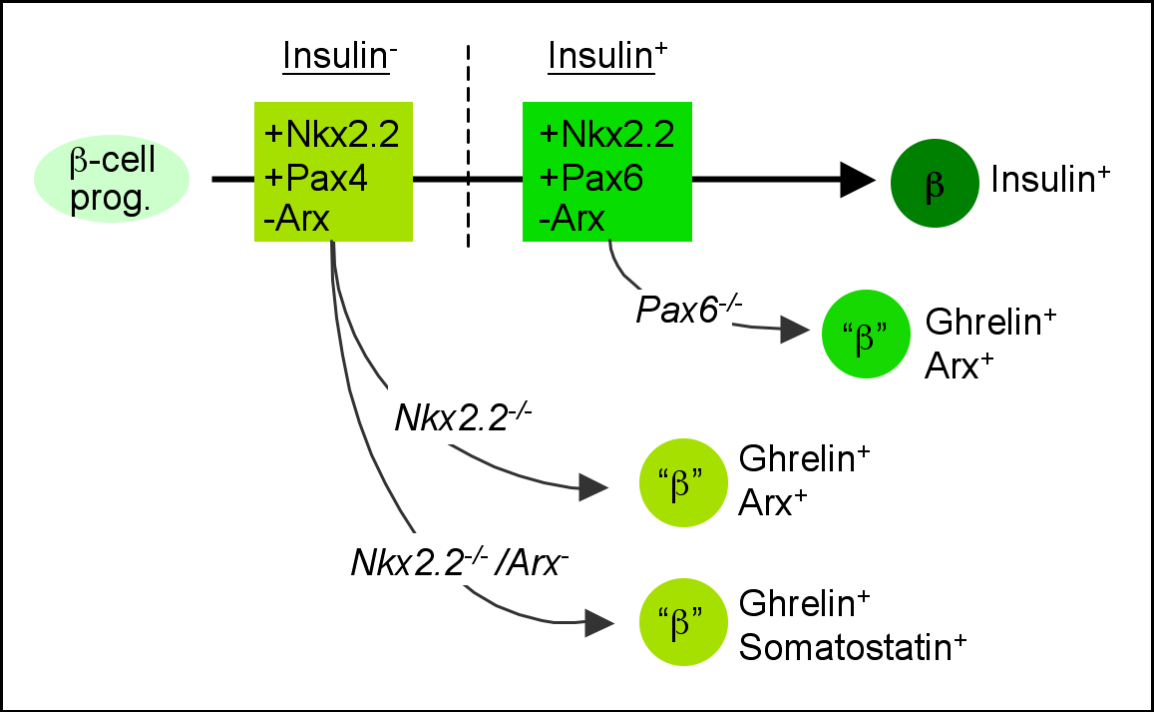
**Schematic representation of transcription factor combinations guiding beta-cell differentiation**. Following the loss of *Nkx2.2 *or *Pax6*, endocrine cells retain some beta-cell characteristics, such as the expression of *iAPP, Pdx1 *and/or *PC1/3 (*indicated as "β"). Rectangles describe transcription factor combinations required to maintain the embryonic beta-cell differentiation program pre- and post-insulin expression (light and dark green, respectively). Shortly after the activation of Ngn3, both Nkx2.2 and Pax4 are necessary for the expression of additional beta-cell specific TF's such as *Nkx6.1 *(not depicted). Concomitantly, this combination of TF's prevents the misexpression of *Arx *and *ghrelin *in committed beta-cells. In the absence of both *Nkx2.2 *and *Arx*, endocrine cells retain the same beta-cell features as noted in the absence of *Nkx2.2 *only, but then express *ghrelin *and *somatostatin*. Pax6 appears necessary for maintaining normal beta-cell identity at later stages of differentiation, probably shortly after *insulin *activation. At that stage Pax6 presumably takes over some of the functions previously exerted by Pax4, whose expression decreases.

Our data indicate that although proper endocrine differentiation/hormone expression is impaired in Pax6- or Nkx2.2-deficient pancreata, committed cells retain some of the characteristics normally associated with mature beta-cells (Figure 8). Our analysis of *Nkx2.2/Arx *single- and double-mutant pancreata allow us, together with the data from others, to conclude that both Pax4 and Arx require the activity of *Nkx2.2 *in order to maintain the transcriptional cascades initiated in early committed beta- and alpha-cell precursors, respectively. Our findings also suggest that one of the coupled functions of Nkx2.2 and Pax4 is the control of *Arx *gene activity in committed beta-cell precursors.

## Methods

### Animals

Mice heterozygous for both *Nkx2.2 *and *Arx *were generated by crossing *Nkx2.2^+/- ^*males [22] with *Arx^+/- ^*females [27] and maintained in an NMRI background. Double heterozygous females were further bred with *Nkx2.2^+/- ^*males. Moreover, we utilized tissue from *Pax6*-nullizygous mice previously described by [24]. Genotyping was performed as described in [22,24,27]. Animal care and experimental use were approved by the Ordnungsamt der Stadt Göttingen, Germany.

### Immunohistochemistry

Pancreata were fixed 30 minutes in 4% paraformaldehyde at 4°C, dehydrated through graded alcohols and embedded in paraffin. 8 μm (P0) or 5 um-sections (E15.5-E17.5) were rehydrated and blocked in PBS containing 10% inactivated fetal calf serum for 60 min. Primary antibodies were diluted in the same medium, applied onto sections, and incubated overnight at 4°C. Slides were washed in PBS and incubated for 45 min with the appropriate secondary antibody diluted in PBS containing 10% inactivated fetal calf serum. Slides were washed in PBS, mounted with DAPI, and viewed by fluorescent or confocal microscopy.

The primary antibodies used were as follows: rabbit anti-Brn4 1/200 [kindly provided by A. Ryan (McGill University, Montreal, QC)]; guinea-pig anti-insulin 1/1000 (Dako); guinea-pig anti-insulin 1/1000 (Linco), guinea-pig anti-glucagon 1/1000 (Linco); rabbit anti-somatostatin 1/600 (Dako); rat anti-somatostatin 1/200 (Millipore); rabbit anti-PP diluted 1/200 (Linco); mouse anti-ghrelin 1/1500 [kindly provided by C. Tomasetto (Université Louis Pasteur, Strasbourg, France)]; goat anti-ghrelin 1/50 (Santa Cruz), rabbit anti-ghrelin 1/1000 (Millipore), rabbit anti-Amylin 1/300 (Phoenix Pharmaceuticals); rabbit anti-Nkx6.1 diluted 1/3000; rabbit anti-Pdx1 diluted 1/1000 [kindly provided by C. Wright (Vanderbilt University, Nashville, TN)]; rabbit anti-Pax6 1/1000 (Covance); rabbit anti-Pax4 1/1000 [kindly provided by B. Sosa-Pineda (St. Jude Children's Research Hospital, Memphis, TN)], rabbit anti-PC1/3 diluted 1/500 (Millipore), rabbit anti-Arx diluted 1/250, guinea-pig anti-Arx diluted 1/500 and rabbit anti-Sox9 1/200 (Millipore).

The secondary antibodies (invitrogen, dilution 1/1000) used for immunofluorescence were as follows: 594-alexa anti-mouse; 488-alexa anti-mouse, 594-alexa anti-rabbit; 488-alexa anti-rabbit; 594-alexa anti-guinea pig; 488-alexa anti-guinea pig; 594-alexa anti-goat and 488-alexa anti-goat. Images were taken using either a Leica TCS confocal-laser scanning microscope or an Olympus BX60 fluorescent microscope.

### Morphometric Analyses

P0 pancreata from wild type, *Arx^-^*, *Nkx2.2^-/- ^*and *Nkx2.2^-/-^Arx^- ^*mice were serially sectioned and every twelfth section (10 sections per pancreas) was stained for insulin, glucagon and ghrelin or somatostatin and ghrelin. Non-overlapping pictures were taken from every section using 10× magnification and same exposure times. Total pancreatic area and fluorescent cell area were determined using the histogram- and colour range functions in Adobe Photoshop. Results are displayed as fluorescent cell area per total pancreatic area. In order to assess the numbers of Arx^+^, Pax4^+ ^(at E16.5) or Nkx6.1^+^Sox9^- ^cells (at E15.5) pancreata were sectioned (5 um) and every eighth section (6 sections per pancreas) was stained with the respective antibodies. The numbers of Arx^+^, Pax4^+ ^or Nkx6.1^+^Sox9^- ^cells were counted by microscopy and subsequently standardized to the pancreatic area.

For both morphometric analysis a single-factor ANOVA coupled to Newman-Keuls test was applied and p-values < 0,05 were assessed as statistically significant compared to wild type (n = 3 pancreata for each genotype). Statistical significance is achieved with the assumption of a normal distribution. In order to determine the percentages of copositive cells (Arx^+^/glucagon^+^, Brn4^+^/ghrelin^+^, Arx^+^/ghrelin^+^, insulin^+^/ghrelin^+^, Pdx1^+^/ghrelin^+^, iAPP^+^/ghrelin^+ ^and PC1/3^+^/ghrelin^+^), 10 microscopy fields (per pancreas) of representative areas were counted. Error bars represent SEM.

### mRNA Quantification

Total RNA was isolated with the RNeasy Plus Mini Kit (Qiagen) using whole pancreata at E16.5 (n = 3 per genotype). 5 ug total RNA of each pancreas was subjected to cDNA synthesis with the SuperScript II Reverse Transcriptase Kit (invitrogen) following the manufacturer's instructions. The amount of Arx (QT00162904), Pax6 (QT01052786) and Nkx2.2 (QT00495502) transcripts was assessed relative to Gusb (QT00176715) using the QuantiTect SYBR Green PCR Kit from Qiagen. Quantitative PCR's were processed using the eppendorf Mastercycler realplex system.

### Glucose levels

Glucose levels were determined with the One Touch Glucose monitoring kit (Johnson & Johnson) using 15 ul of peripheral blood.

## Competing interests

The authors declare that they have no competing interests.

## Authors' contributions

SK designed the study, carried out all experiments, and wrote the manuscript. PC, AM and PS conceived the study, and participated in its design and coordination and helped to draft and write the manuscript. All authors read and approved the final manuscript.
